# Oxalate Nephropathy: A Case Report of Acute Kidney Injury Due to Juice Diet

**DOI:** 10.7759/cureus.51226

**Published:** 2023-12-28

**Authors:** Niranjan Raja, Hemachandar Radhakrishnan, Sivasankar Masilamani

**Affiliations:** 1 Department of Nephrology, Mahatma Gandhi Medical College and Research Institute, Puducherry, IND

**Keywords:** juice diet, hyperoxaluria, vitamin c, acute kidney injury, oxalate nephropathy

## Abstract

Oxalate nephropathy occurs due to the deposition of calcium oxalate crystals in kidney tubules and/or the interstitium as a part of primary or secondary hyperoxaluria. Secondary oxalate nephropathy can occur even with moderately high doses of ascorbic acid intake under yet unidentified clinical circumstances. Vitamin C, although traditionally considered an antioxidant, leads to the formation of superoxide and subsequent generation of reactive oxidant species at pharmacologic concentrations. Ascorbic acid is partly converted to oxalic acid, which is responsible for deposition and renal tubular injury.

We report a case of a diabetic patient with normal kidney function who was put on a juice diet for a week due to upper gastrointestinal bleeding. He developed acute kidney injury due to biopsy-proven oxalate nephropathy requiring dialysis. Though he was lost to follow-up after two weeks on dialysis, he was expected to have only a slow recovery or become dependent on dialysis given his age, comorbidities, and extent of tubular involvement. Hence, caution should be exercised before supplementing vitamin C either in its natural form or as a drug. Risk factors for secondary oxalate nephropathy due to excessive intake of oxalate or its precursor are likely to be age, diabetes, dehydration, and underlying chronic kidney disease. Most of the patients do not have a complete recovery of kidney function, and many become dependent on dialysis.

## Introduction

Oxalate nephropathy occurs due to the deposition of calcium oxalate crystals in kidney tubules and/or the interstitium. Oxalate nephropathy can occur as a part of primary or secondary hyperoxaluria. Primary hyperoxaluria is a group of disorders with an autosomal recessive inheritance of enzymatic deficiencies such as alanine:glyoxylate aminotransferase, glyoxylate reductase/D-glycerate dehydrogenase, and 4-hydroxy-2-oxoglutarate aldolase, leading to primary hyperoxaluria types 1, 2, and 3, respectively [1]. Secondary hyperoxaluria is relatively more common and can occur due to increased dietary oxalate or oxalate precursor intake, increased intestinal oxalate availability, decreased intestinal oxalate degradation, or increased colonic permeability to oxalate [2].

Increased dietary oxalate or its precursor intake has been reported in the literature, especially due to excessive ingestion of vegetable and/or fruit juices [3]. Increased intestinal oxalate availability and colonic permeability to oxalate occur in enteric hyperoxaluria, which is a consequence of conditions such as inflammatory bowel disease, bowel surgeries, pancreatitis, and the use of orlistat [2]. Decreased intestinal oxalate degradation can occur due to alterations in the gut microbiota more commonly seen with antibiotics. *Oxalobacter formigenes* is a gram-negative anaerobic bacterium in the normal gut microbiota. The depletion of gut *Oxalobacter* has been associated with increased urinary oxalate in kidney stone-forming patients [4]. Here, we report a case of a patient who was admitted for upper gastrointestinal bleeding and subsequently kept on a juice diet, leading to acute kidney injury.

## Case presentation

An 85-year-old man presented to the Department of Nephrology with bilateral pedal edema up to the knees and oliguria for two days. He had been diabetic for three years and had been on metformin and glipizide. He was neither an alcoholic nor a smoker and had no family history of kidney disease. Two days prior to presentation, he left a hospital at his request where he was treated for upper gastrointestinal bleeding. He had four to six episodes of hematemesis containing approximately 100 mL each with melena and was admitted in a hemodynamically stable state and was kept nil per os for a day. Serial monitoring of hemoglobin showed a drop of 1.5 g/dL for which one unit of packed red blood cells (350 mL) was transfused. Abdominal computed tomography (CT) was normal except for a liver cyst measuring 1.3 x 0.8 cm cyst and an exophytic simple cyst in the upper pole of the left kidney measuring 5.2 x 4.5 cm. An upper gastrointestinal endoscopy was performed, which revealed a gastric ulcer treated with proton pump inhibitors. Though he was advised a semi-solid diet 48 hours after the endoscopy, he preferred a liquid diet for a week. During the course in that hospital, he remained hemodynamically stable but his kidney function deteriorated, with an admission creatinine of 1.12 mg/dL to a peak creatine of 5.43 mg/dL taken on the 11th day after admission. Ultrasonography of the abdomen revealed normal-sized kidneys with slightly increased echoes and a large exophytic cyst in the upper pole of the left kidney without any features of obstruction.

**Figure 1:**
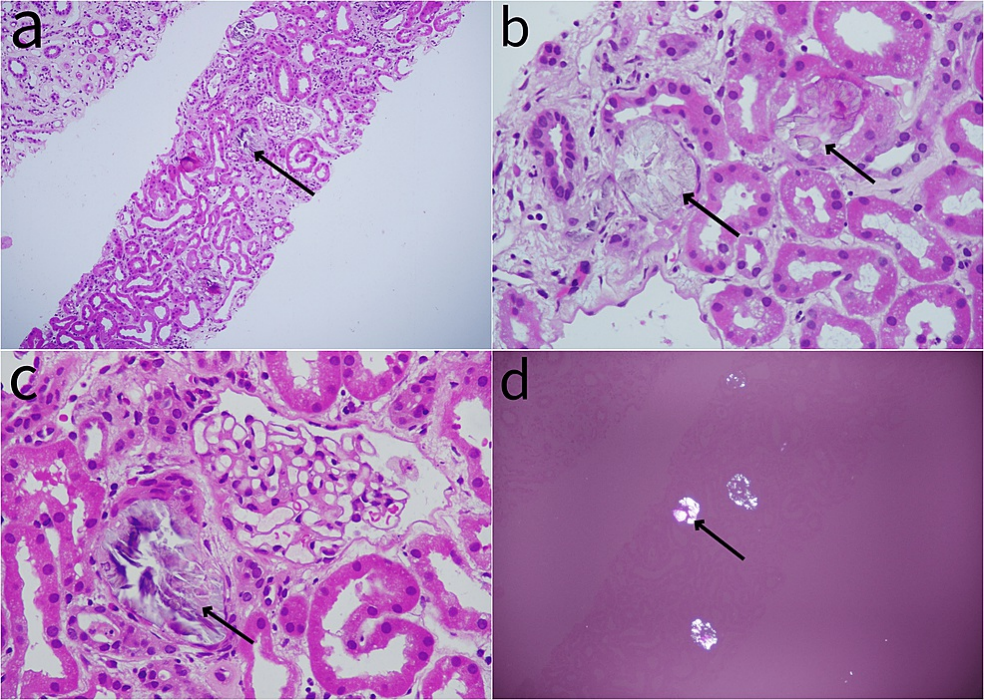
Renal histology with calcium oxalate crystals under light microscopy. (a) A low-power view (10x) shows diffuse tubular degenerative changes with numerous intracellular and intraluminal tubular calcium oxalate deposits and the most prominent tubule affected in the slide indicated by the arrow (H&E). (b) At high magnification, the calcium oxalate deposits form intraluminal translucent crystals seen in two tubules indicated by arrows along with changes of acute tubular necrosis (H&E). (c) The same field as (a) at a higher magnification with the arrow indicating the intratubular polyhedral calcium oxalate crystals (H&E). (d) Calcium oxalate crystals are shown as birefringent under polarized light (black arrow). H&E, hematoxylin and eosin

On admission to our hospital, the patient denied taking any painkillers or native medications and was initiated on dialysis. Urinalysis revealed 1+ proteinuria, 3-5 red blood cells/high power field, 15-20 pus cells/high power field, and granular casts. His urine protein-to-creatinine ratio was 1.8. Hepatitis B surface antigen, Hepatitis C viral antibody, HIV serology, and antineutrophilic cytoplasmic antibodies were negative, and complement 3 and complement 4 levels were normal. Subsequently, a kidney biopsy was performed, which revealed that approximately 20-25% of the tubules contained colorless fan-shaped crystals that were birefringent under polarized light and tubular epithelial cell injury (Figure 1). Out of 11 glomeruli present in the biopsy, three were globally sclerotic, and the viable glomeruli were normocellular with patent capillary loops. A repeat CT of the abdomen in our hospital did not show nephrocalcinosis, and his 24-hour urine oxalate was 13.57 mg (normal: 7-44). The urine routine test did not reveal calcium oxalate crystals. On further questioning, he revealed that he was taking only pomegranate juice without added sugar three to four times daily, and, on an occasion, sweet lime juice, for a week during his admission to the previous hospital. He was advised to consume low oxalate content food and avoid juices and was started on Oxalobacter formigenes capsules twice daily. After two weeks on dialysis, he failed to show up for dialysis and was lost to follow-up.

## Discussion

**Figure 2:**
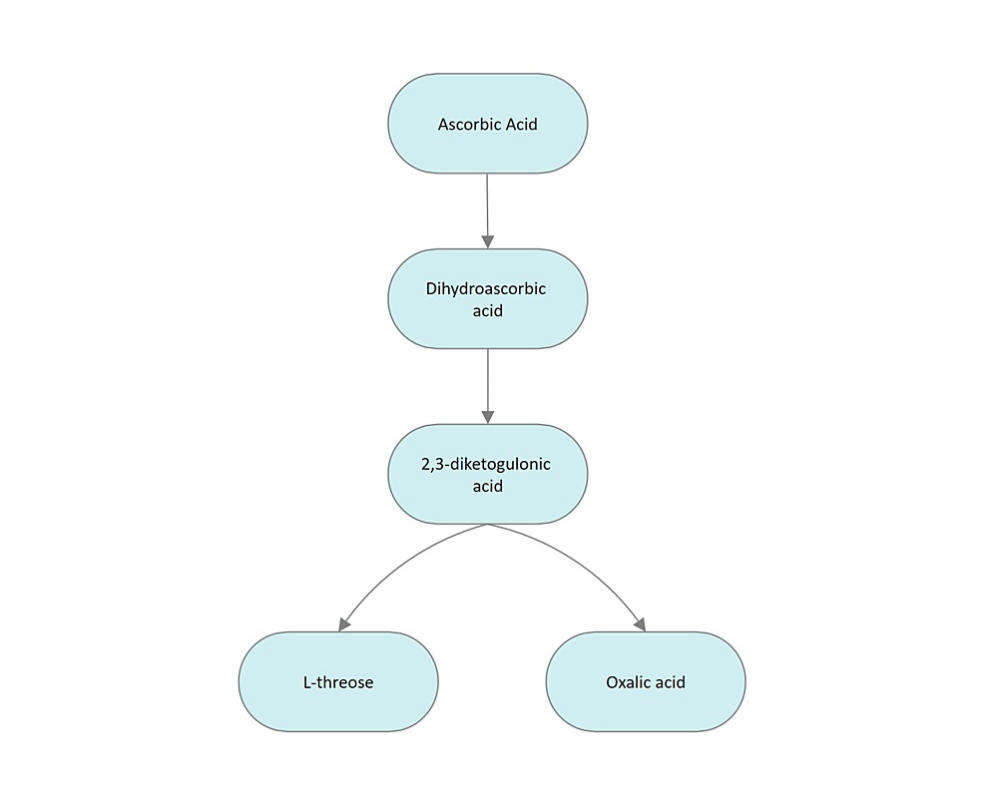
Metabolism of ascorbate to oxalic acid Image Credits: Niranjan Raja

Vitamin C (ascorbic acid) cannot be synthesized by humans due to mutations in the L-gulono-γ-lactone oxidase (*GLO*) gene, which codes for the enzyme responsible for catalyzing the last step of vitamin C biosynthesis [5]. Vitamin C is usually promoted as an antioxidant, as it can reduce oxidized species, but this terminology may be misleading. Electrons from ascorbate can reduce metal ions, leading to the formation of superoxide and subsequent generation of reactive oxidant species at pharmacologic concentrations [6,7]. Vitamin C is partly converted to oxalic acid (Figure 2), which is responsible for deposition and renal tubular injury. Authors can only speculate that primates with a wide variety of food habits have the inactivated *GLO* gene as a part of natural selection to reduce the incidence of secondary oxalate nephropathy.

Many reports describe oxalate nephropathy after the administration of vitamin supplements or citrus juice [8-10]. The source of vitamin C in our patient was pomegranate juice, which contains approximately 52.8 to 72.0 mg/100 g fresh weight of seeds [11]. Our patient took approximately 600-900 mL of fresh pomegranate juice per day. He was estimated to have consumed approximately 450 mg of vitamin C per day for a week, while the recommended dietary allowance is 90 mg/day for adult men and 75 mg/day for adult women. Friedman et al. described a similar case of oxalate nephropathy with ascorbate supplementation at a dosage of 500 mg/day for four weeks [12]. In a case series by Bakul et al, two patients developed oxalate nephropathy by ingestion of Averrhoa bilimbi (Irumban Puli), which is rich in oxalate, for four to five days [13], while Nair et al. describe a similar case even after a one-time ingestion of the Irumban Puli juice of around 100 mL for its medicinal benefits [14].

Our patient was 85 years old and had diabetes mellitus. Age appears to be an important risk factor in secondary oxalate nephropathy, as demonstrated by a mean age of 56.4 years in a systematic review by Lumlertgul et al. [2] and above 60 years in four large-scale case series [15-18]. Nearly one-third of patients reported in these studies had diabetes. Because diabetics excrete more oxalate than other people, if this a risk factor for oxalate nephropathy or a mere association of the elderly population is yet to be confirmed.

Our patient had a 24-hour urine oxalate within the normal range, as shown in a systematic review on secondary oxalate nephropathy, and only 26% of the patients had high urine oxalate levels [2]. Moreover, it is likely that oxalate excretion may have gone as the glomerular filtration rate went down. Furthermore, it was tested in our patient only 10 days after stopping juices. Fruit juices and citrate supplementation are recommended for kidney stone formers to correct hypocitraturia, a risk factor for calcium stones [19]. Furthermore, pomegranate juice has been shown to inhibit oxalate stone formation in animals due to its antioxidant properties by reducing reactive oxygen species, inducible nitric oxide synthase, selective nuclear factor-kB, p38-mitogen-activated protein kinase, and transcription factors such as nuclear factor kappa-B [20]. It is likely that our patient developed acute kidney injury due to oxalate deposition and acute tubular necrosis further worsened by volume depletion. Moderation in dose might be the key to health benefits.

Although our patient was lost to follow-up, he was expected to have only a slow recovery or become dependent on dialysis given his age, comorbidities, and extent of tubular involvement.

## Conclusions

Oxalate nephropathy can occur even with moderately high doses of ascorbic acid intake including a juice diet, as seen in our patient. Our patient was expected to have only a slow recovery of kidney function or become dependent on dialysis given his age and comorbidity of diabetes. This report highlights that secondary oxalate nephropathy can occur even with an unsuspicious juice diet, and caution should be exercised before supplementing vitamin C either in its natural form or as a drug.

## References

[1] Demoulin N, Aydin S, Gillion V, Morelle J, Jadoul M (2022). Pathophysiology and management of hyperoxaluria and oxalate nephropathy: a review. Am J Kidney Dis.

[2] Lumlertgul N, Siribamrungwong M, Jaber BL, Susantitaphong P (2018). Secondary oxalate nephropathy: a systematic review. Kidney Int Rep.

[3] Barman AK, Goel R, Sharma M, Mahanta PJ (2016). Acute kidney injury associated with ingestion of star fruit: acute oxalate nephropathy. Indian J Nephrol.

[4] Tasian GE, Jemielita T, Goldfarb DS, Copelovitch L, Gerber JS, Wu Q, Denburg MR (2018). Oral antibiotic exposure and kidney stone disease. J Am Soc Nephrol.

[5] Drouin G, Godin JR, Pagé B (2011). The genetics of vitamin C loss in vertebrates. Curr Genomics.

[6] Parrow NL, Leshin JA, Levine M (2013). Parenteral ascorbate as a cancer therapeutic: a reassessment based on pharmacokinetics. Antioxid Redox Signal.

[7] Podmore ID, Griffiths HR, Herbert KE, Mistry N, Mistry P, Lunec J (1998). Vitamin C exhibits pro-oxidant properties. Nature.

[8] McHugh GJ, Graber ML, Freebairn RC (2008). Fatal vitamin C-associated acute renal failure. Anaesth Intensive Care.

[9] Rathi S, Kern W, Lau K (2007). Vitamin C-induced hyperoxaluria causing reversible tubulointerstitial nephritis and chronic renal failure: a case report. J Med Case Rep.

[10] Alkhunaizi AM, Chan L (1996). Secondary oxalosis: a cause of delayed recovery of renal function in the setting of acute renal failure. J Am Soc Nephrol.

[11] Opara LU, Al-Ani MR, Al-Shuaibi YS (2009). Physico-chemical properties, vitamin C content, and antimicrobial properties of pomegranate fruit (Punica granatum L.). Food Bioprocess Technol.

[12] Friedman AL, Chesney RW, Gilbert EF, Gilchrist KW, Latorraca R, Segar WE (1983). Secondary oxalosis as a complication of parenteral alimentation in acute renal failure. Am J Nephrol.

[13] Bakul G, Unni VN, Seethaleksmy NV (2013). Acute oxalate nephropathy due to 'Averrhoa bilimbi' fruit juice ingestion. Indian J Nephrol.

[14] Nair S, George J, Kumar S, Gracious N (2014). Acute oxalate nephropathy following ingestion of Averrhoa bilimbi juice. Case Rep Nephrol.

[15] Buysschaert B, Aydin S, Morelle J, Gillion V, Jadoul M, Demoulin N (2020). Etiologies, clinical features, and outcome of oxalate nephropathy. Kidney Int Rep.

[16] Yang Y, Sharma PD, Nair V (2020). Kidney oxalate crystal deposition in adult patients: a relatively common finding. Clin Nephrol.

[17] Nasr SH, D'Agati VD, Said SM, Stokes MB, Largoza MV, Radhakrishnan J, Markowitz GS (2008). Oxalate nephropathy complicating Roux-en-Y gastric bypass: an underrecognized cause of irreversible renal failure. Clin J Am Soc Nephrol.

[18] Cartery C, Faguer S, Karras A (2011). Oxalate nephropathy associated with chronic pancreatitis. Clin J Am Soc Nephrol.

[19] Phillips R, Hanchanale VS, Myatt A, Somani B, Nabi G, Biyani CS (2015). Citrate salts for preventing and treating calcium containing kidney stones in adults. Cochrane Database Syst Rev.

[20] Ilbey YO, Ozbek E, Simsek A, Cekmen M, Somay A, Tasci AI (2009). Effects of pomegranate juice on hyperoxaluria-induced oxidative stress in the rat kidneys. Ren Fail.

